# Supplementary material for: Moderate to severe acute pain disturbs motor cortex intracortical inhibition and facilitation in orthopedic trauma patients: A TMS study
Source: PLoS One. 2020 Mar 20;15(3):e0226452. doi: 10.1371/journal.pone.0226452 (PMC7083311; doi:10.1371/journal.pone.0226452)
Supplement: Attachment — Submitted filename: PLOS One response to Reviewers.docx [file pone.0226452.s003.docx]

Reviewer #1

**Comment #1**: In regard to contamination of SICI by SICF, I was not suggesting to use AMT. The issue could have been accounted for by using a lower %RMT conditioning stimulus. I understand why the authors would want to include the intensity commonly tested within the existing literature, but inclusion of an additional, lower intensity, conditioning stimulus would have been very feasible. At the very least, the possibility of SICF contamination should be addressed to some degree in the discussion.

**Response to Comment #1**: We have addressed this comment in the limitation section.

**Comment #2:** The authors did not address why they elected to retain outcomes of all post-hoc comparisons in the figures, despite the fact that they’re reported in the text (see comment 9).

**Response to Comment #2**: Our apologies. We have made the necessary changes and removed all results from the post-hoc statistics.

**Comment #3**: Typos on line 224 (RMT criteria still refer to 0.5mV MEP, which should be 0.05mv) and 243 (LICI stimuli referred to as subthreshold, should be suprathreshold).

**Response to comment #3:** Thank you for picking that up. We have made the necessary changes.
